# Correction: Arossa et al. Differential Responses to Heat Stress Between Freshly Isolated and Long-Term Cultured *Symbiodinium*. *Microorganisms* 2026, *14*, 455

**DOI:** 10.3390/microorganisms14051078

**Published:** 2026-05-11

**Authors:** Silvia Arossa, Shannon Grace Klein, Jacqueline Victoria Alva Garcia, Alexandra Steckbauer, Naira Pluma, Luca Genchi, Sergey P. Laptenok, Shiou-Han Hung, Octavio R. Salazar, Manuel Aranda, Carlo Liberale, Carlos Manuel Duarte

**Affiliations:** 1 Biological and Environmental Science and Engineering Division (BESE), King Abdullah University of Science and Technology (KAUST), Thuwal 23955-6900, Saudi Arabia; shannon.klein@kaust.edu.sa (S.G.K.); jacqueline.alvagarcia@kaust.edu.sa (J.V.A.G.); alexandra.steckbauer@kaust.edu.sa (A.S.); luca.genchi@kaust.edu.sa (L.G.); siarhei.laptenok@kaust.edu.sa (S.P.L.); shiou-han.hung@kaust.edu.sa (S.-H.H.); octavio.salazarmoya@kaust.edu.sa (O.R.S.); manuel.aranda@kaust.edu.sa (M.A.); carlo.liberale@kaust.edu.sa (C.L.); carlos.duarte@kaust.edu.sa (C.M.D.); 2 National Center for Wildlife (NCW), P.O. Box 61681, Riyadh 11575-4508, Saudi Arabia; 3 Institut Fresnel, CNRS, Centrale Med, Aix-Marseille University, 13284 Marseille, France; 4 Computer, Electrical and Mathematical Sciences and Engineering (CEMSE), King Abdullah University of Science and Technology (KAUST), Thuwal 23955-6900, Saudi Arabia

In the original publication [1], there were mistakes in Figures 1–3 as published.

The y-axis label for Figure 1A should be dO_2_ (mg L^−1^). The y-axis label for Figure 1B should be *p*CO_2_ (µatm). The corrected Figure 1 and its legend appear below.

The x-axis label for Figure 2E should be “fresh” and “long-term cultured”. The color assignments in the figure legend were incorrect. The corrected Figure 2 and its legend appear below.

The labels for Figure 3B should be LT Day and LT Night. The color assignments in the figure legend were incorrect. The corrected Figure 3 and its legend appear below.

The authors state that the scientific conclusions are unaffected. This correction was approved by the Academic Editor. The original publication has also been updated.

## Figures and Tables

**Figure 1:**
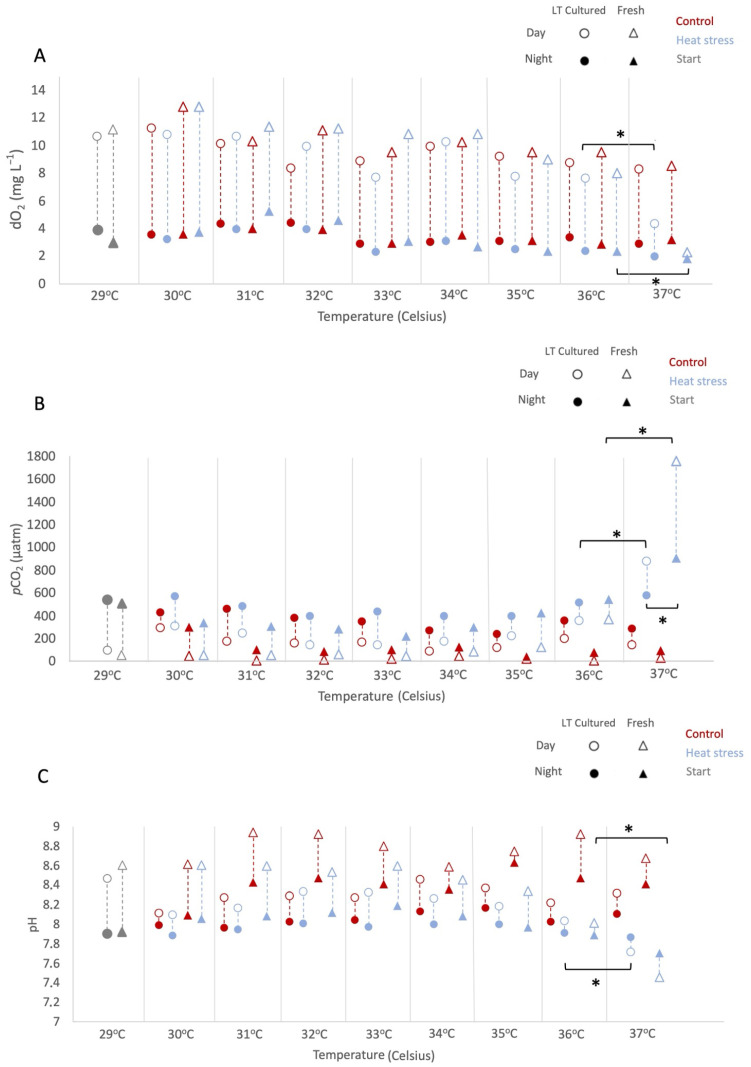
Temporal variations in environmental parameters for “long-term cultured” and “freshly isolated” *Symbiodinium* cultures under thermal stress and control temperature. Temporal changes in (**A**) dissolved oxygen (mg L^−1^), (**B**) *p*CO_2_ (µatm), and (**C**) pH in *Symbiodinium* cultures. Day values are indicated by unfilled markers, while night values are shown with filled markers. Control samples (kept at 29 °C throughout) are denoted in red, ramping samples (subjected to +1 °C per day) in blue, and initial values in gray. Circles represent “long-term cultured” cultures; (“LT cultured” in the figure) triangles represent “freshly isolated” cultures (“fresh” in the figure). Error bars are not reported (see Supplementary Materials). Significant differences at *p* < 0.05 are represented as “*”. In all figures, the control group was maintained at a constant temperature of 29 °C throughout the entire experiment. However, on the x-axis, different temperatures are indicated to represent the conditions under which the ramping samples were exposed on specific days. This implies that the control group maintained a consistent temperature regardless of the temperature variations depicted on the x-axis for the ramping samples.

**Figure 2:**
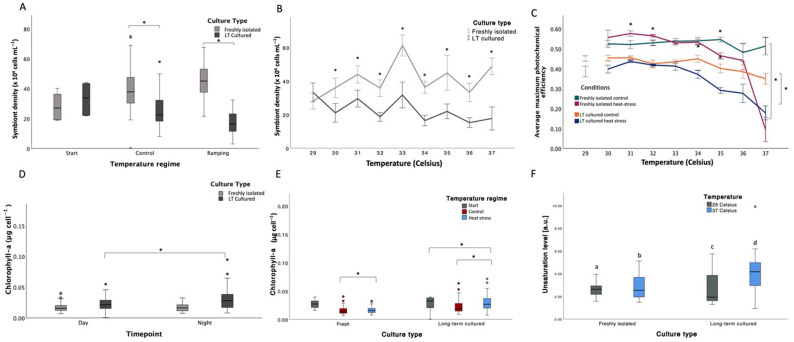
Variations in productivity-related responses of *Symbiodinium* during a thermal stress experiment (non-transformed data). (**A**) Symbiont density (cells × 10^4^ mL^−1^) plotted by temperature regime and culture type (“freshly isolated” in gray and “long-term cultured” in dark gray; see Table S23 for statistical details). (**B**) Symbiont density (cells × 10^4^ mL^−1^) plotted by temperature and culture type (“freshly isolated” in gray and “long-term cultured” in black; see Table S24 for statistical details). (**C**) Average maximum photochemical efficiency (Fv/Fm) plotted by temperature and conditions (unlinked points with gray bars: start; dark green: “freshly isolated” control; purple: “freshly isolated” under heat stress; orange: “long-term cultured” control; dark blue: “long-term cultured” under heat stress). (**D**) Chlorophyll-a (µg cell^−1^) plotted by timepoint and culture type (“freshly isolated” in gray and “long-term cultured” in dark gray). (**E**) Chlorophyll-a (µg cell^−1^) plotted by culture type (“freshly isolated” and “long-term cultured”) and temperature regime (start in gray, control in red, heat stress in dark blue). (**F**) Unsaturation levels (a.u.) of thylakoid membrane lipids plotted by culture type (“freshly isolated” and “long-term cultured”) and temperature (29 °C in gray and 37 °C in blue). Different letters indicate significant pairwise differences (post hoc tests, *p* < 0.05). In all panels, whiskers indicate the full range (minimum to maximum), boxes represent the interquartile range, and the horizontal line indicates the median. Circles represent outliers. In all graphs, “long-term cultured” is abbreviated as “LT cultured” to shorten labels. Significant differences identified on transformed data are indicated by asterisks (*, *p* < 0.05); asterisks represent pairwise comparisons between culture types within temperature regimes (panel (**A**); Table S23) or at individual temperatures (panel (**B**); Table S24). In panels where responses are plotted against temperature, control cultures were maintained at a constant temperature of 29 °C throughout the experiment, and temperatures shown on the x-axis represent the conditions experienced by ramping samples on specific days; thus, control cultures remained at 29 °C despite the temperature values shown for ramping treatments.

**Figure 3:**
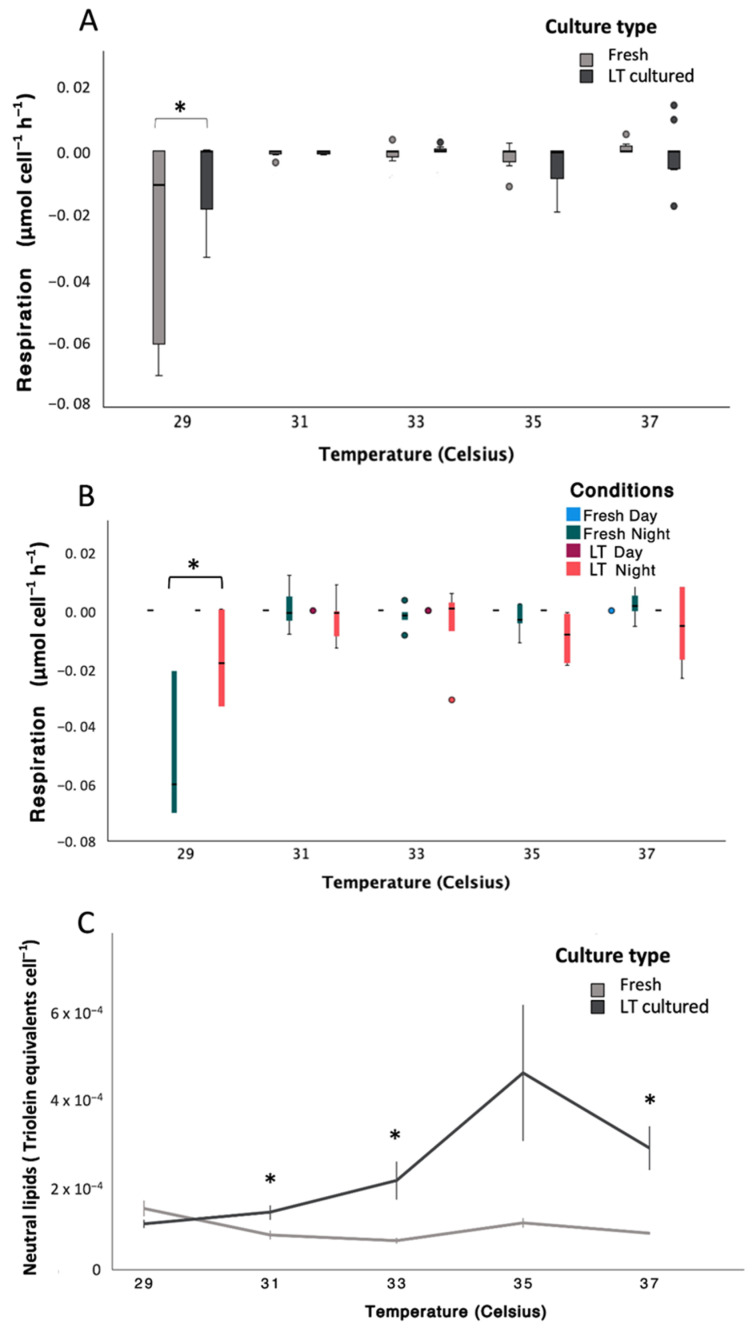
Variations in metabolic and energy storage responses of *Symbiodinium* during a thermal stress experiment (non-transformed data). (**A**) Respiration (µmol cell^−1^ h^−1^) plotted by temperature and culture type (“freshly isolated” in gray and “long-term cultured” in black; see Tables S35 and S36 for statistical details). (**B**) Respiration (µmol cell^−1^ h^−1^) plotted by temperature and conditions (“freshly isolated” day in light blue, “freshly isolated” night in dark green, “long-term cultured” day in dark red, and “long-term cultured” night in light red; see Tables S35 and S38 for statistical details). (**C**) Neutral lipid content (triolein equivalents cell^−1^) plotted by temperature and culture type (“freshly isolated” in gray and “long-term cultured” in black; see Tables S41 and S42 for statistical details). In all panels, whiskers indicate the full range (minimum to maximum), boxes represent the inter-quartile range, and the horizontal line indicates the median. Circles represent outliers. In all graphs, “long-term cultured” is abbreviated as “LT cultured” or “LT” to shorten labels. Significant differences identified on transformed data are indicated by asterisks (*, *p* < 0.05). In panels where responses are plotted against temperature, control cultures were maintained at a constant temperature of 29 °C throughout the experiment, and temperatures shown on the x-axis represent the conditions experienced by ramping samples on specific days; thus, control cultures remained at 29 °C despite the temperature values shown for ramping treatments.
